# Disc Height and Angle Changes on Radiographs and Magnetic Resonance Imaging after Anterior or Posterior Percutaneous Endoscopic Cervical Discectomy

**DOI:** 10.3390/jcm13123414

**Published:** 2024-06-11

**Authors:** Chun-Pi Chang, Hsi-Kai Tsou, Wen-Hsien Chen, Ting-Hsien Kao, Chih-Wei Huang, Chung-Yuh Tzeng, Tse-Yu Chen, Ruei-Hong Lin

**Affiliations:** 1 Department of Neurosurgery, Neurological Institute, Taichung Veterans General Hospital, Taichung 40705, Taiwan; 2 Department of Surgery, Zuoying Armed Forces General Hospital, Kaohsiung 81345, Taiwan; 3 Functional Neurosurgery Division, Neurological Institute, Taichung Veterans General Hospital, Taichung 40705, Taiwan; 4 Department of Post-Baccalaureate Medicine, College of Medicine, National Chung Hsing University, Taichung 402202, Taiwan; 5 Department of Rehabilitation, Jen-Teh Junior College of Medicine, Nursing and Management, Miaoli 356006, Taiwan; 6 College of Health, National Taichung University of Science and Technology, Taichung 403027, Taiwan; 7 Department of Radiology, Taichung Veterans General Hospital, Taichung 40705, Taiwan; 8 Department of Industrial Engineering and Enterprise Information, Tunghai University, Taichung 407224, Taiwan; 9 Department of Orthopedics, Taichung Veterans General Hospital, Taichung 40705, Taiwan; 10 Department of Medicinal Botanicals and Foods on Health Applications, Da-Yeh University, Changhua 515006, Taiwan; 11 Institute of Biomedical Sciences, National Chung Hsing University, Taichung 402202, Taiwan; 12 Ph.D. Program in Translational Medicine, National Chung Hsing University, Taichung 402202, Taiwan; 13 Ph.D. Program in Tissue Engineering and Regenerative Medicine, National Chung Hsing University, Taichung 402202, Taiwan

**Keywords:** cervical disc herniation, cervical disc height, percutaneous endoscopic cervical discectomy, posterior percutaneous endoscopic cervical discectomy, anterior percutaneous endoscopic cervical discectomy

## Abstract

**Objectives**: Cervical disc herniation (CDH) leads to pain, numbness, and potential disability. Percutaneous endoscopic cervical discectomy (PECD) offers an anterior or posterior approach. This study aims to compare postoperative disc height and angle changes one year after PECD, considering both approaches. **Methods**: We retrospectively reviewed the data from patients with CDH who underwent PECD from October 2017 to July 2022. Cervical disc height was measured using the preoperative and one-year postoperative magnetic resonance imaging (MRI) examinations. Lordotic angle (LA), global alignment angle (GAA), segmental alignment angle (SAA), and slippage distance (SD) at the surgical level were measured on radiographs in the neutral, flexion, and extension positions. **Results**: Thirty-eight patients who underwent posterior PECD (PPECD) and five patients who underwent anterior PECD (APECD) were included in the evaluation. The mean age of the patients was 47.4 years (range: 29–69 years). There was a significant difference in the preoperative and one-year postoperative GAA and SAA in extension in the PPECD group (*p* = 0.003 and 0.031, respectively). The mean decreased disc height one-year postoperative was 1.30 mm in the APECD group and 0.3 mm in the PPECD group by MRI. A significant disc height decrease was observed in the APECD group (*p* < 0.001). **Conclusions**: Treating CDH with PPECD or APECD is feasible, as it can relieve symptoms and reduce disability. Stability remained unaffected during the first year after surgery, even though there was an increase in angulation during extension. Despite a significant decrease in disc space following APECD, patients reported significant symptom improvement and no new symptoms.

## 1. Introduction

Persons with symptomatic compression of cervical nerve roots or the spinal cord may present with symptoms such as radiating pain, unusual sensations in specific dermatomes, and/or muscle weakness. The compression often results from cervical disc herniation (CDH) or the formation of osteophytes, both of which contribute to neuroforaminal narrowing or spinal stenosis. These conditions can occur as a result of degenerative changes or traumatic injuries.

Anterior decompression and fusion have traditionally been the standard treatment approach for CDH, but posterior foraminotomy has emerged as a common alternative. In addition to treating lateral disc herniation, posterior foraminotomy can also be used to manage neuroforaminal stenosis [1]. Numerous reports have documented excellent clinical outcomes with minimal complications associated with percutaneous endoscopic cervical discectomy (PECD) [2,3,4,5,6]. Endoscopic cervical foraminotomy or discectomy has emerged as a prominent alternative for the management of cervical neuroforaminal stenosis [7,8,9,10,11].

Advances in technology and the development of specialized instruments, such as endoscopes and endoscopic surgical tools, have expanded the use of endoscopic techniques to more complex cases. Since the early 2000s, PECD has gained recognition as a viable surgical intervention for the management of CDH. Initially, PECD, denoted percutaneous endoscopic discectomy, was performed via an anterior cervical trans-discal approach. In 2007, Ruetten et al. introduced posterior PECD (PPECD), which has become an increasingly popular approach. Currently, it is essential to differentiate the approaches of PECD as either anterior PECD (APECD) or PPECD [2,7].

The choice of surgical approach is determined by the specific location of the pathology. An anterior cervical approach is generally preferred for the removal of central disc herniation. However, patients with certain conditions such as cervical segmental instability, anterior angulation greater than 11°, intraforaminal or extraforaminal CDH with calcification, anterior disc height < 4 mm, anterior osteophytes, sequestration, or significant myelopathy (Nurick grade 3 or higher) are not considered suitable candidates for an anterior approach [12]. Persistent or intolerable radicular pain and/or neurological deficits resulting from CDH, foraminal stenosis, or zygapophyseal joint cysts compressing cervical nerves, as well as residual pathologies in the anterior foraminal region following anterior surgery, and rare posterior pathologies such as bleeding, localized abscesses, and cysts can be effectively managed using a posterior approach [13].

Few studies have compared APCED and PPECD for the treatment of CDH. Yang et al. reported that the anterior approach was associated with an increased volume of disc removal and larger decrease in the postoperative intervertebral vertical height [14]. However, the impact of this procedure on stability or range of motion (ROM) was not discussed.

In this report, we present the outcomes of 43 patients with symptomatic CDH treated with either APECD or PPECD, with a minimum follow-up period of one year.

## 2. Materials and Methods

### 2.1. Patient Information

The records of 43 consecutive patients with symptomatic CDH treated between October 2017 and July 2022 were retrospectively reviewed. There were 26 males and 17 females, and three patients had undergone previous surgery at different levels. One patient who was treated with APECD had received cervical disc arthroplasty (CDA) at another hospital. Of the patients treated with PPECD, two patients had undergone different procedures; one patient had CDA and the other patient received anterior cervical discectomy and fusion (ACDF) from the same neurosurgeon (HK Tsou). Patients with CDH where the main part of the herniation was located medially to the lateral spinal cord edge was considered an indication for an anterior approach. On the other hand, a posterior approach was considered more appropriate for intra- or extraforaminal CDH. Of the 43 patients, five received APECD and 38 received PPECD. In total, there were four instances of calcified discs and eight of ruptured discs. In the PPECD group, the shoulder approach was used in 14 patients, and the axillary approach was used in 24 patients.

### 2.2. Patient Selection

Inclusion criteria for the study were: (1) received continuous conservative treatment for at least 6 weeks; (2) neurological symptoms such as radiculopathy and/or myelopathy, with corresponding imaging showing CDH with/without foraminal stenosis; and (3) a ventral intervertebral height of ≥4 mm to be considered for APECD. Exclusion criteria were: (1) cervical instability or deformity; (2) ossification of the posterior longitudinal ligament; (3) an anterior intervertebral height < 4 mm to be considered for APECD; (4) previous surgery at the same segment; and (5) surgery not feasible due to systemic disease or mental disorder.

### 2.3. Clinical and Radiological Parameters

Patient demographic and clinical information was obtained by reviewing their medical records. Radiographic images were taken at various time points, including preoperative and 3 months and 1 year postoperative. An assessment of the patient data was conducted by two experienced neurosurgeons, and validated by an experienced neuroradiologist.

**Figure 1:**
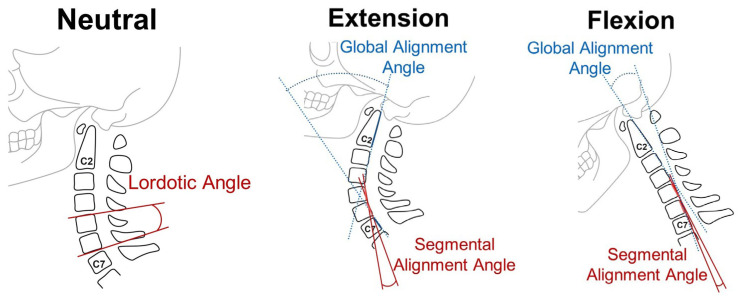
Schematic drawings showing lateral radiographic measurements in the neutral, extension, and flexion positions.

Radiographic measurements were taken from lateral radiographs taken in flexion, extension, and a neutral position. The lordotic angle (LA, or functional spinal unit angle), global alignment angle (GAA), segmental alignment angle (SAA), and slippage distance (SD) of the index level (surgical level) were measured using methods previously described [15,16,17]. Global and segmental ROM were calculated by determining the difference in alignment angle between the flexion and extension positions on lateral radiographs, which allowed for an assessment of dynamic changes in alignment at the index level. The slippage distance was measured on lateral radiographs taken in extension and flexion [18] (Figure 1). Measurement involved assessing the amount of translation or displacement of the vertebral body at the index level between these two positions. In this study, patients with herniation at the C6–C7 or C7–T1 levels were included in the analysis if their lateral radiography images were clear and could be assessed. Patients were excluded if the lateral radiographs were obstructed by the shoulder, thus making it difficult to obtain accurate measurements. The intervertebral disc space was measured based on magnetic resonance imaging (MRI) sagittal views before and one year after the operation. To reduce measurement bias, the anterior, middle, and posterior disc heights were measured on the MRI, and the average of the three measurements was calculated and used in the analysis.

### 2.4. Preoperative Workup, Positioning, and Operative Technique

**Figure 2:**
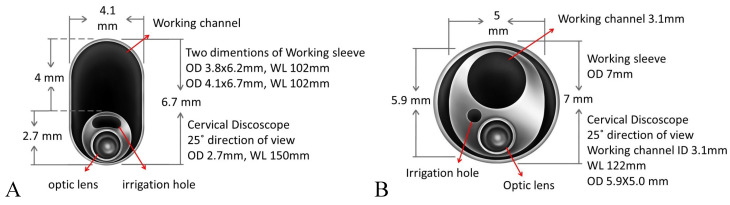
The endoscope for (**A**) APECD and (**B**) PPECD.

The preoperative workup included plain radiography, MRI, and neurological and electrophysiological studies with electromyograph (EMG) and measurement of nerve conduction velocity (NCV). If necessary, the operation site was shaved. All patients received a single dose of antibiotics for prophylaxis, according to our standard protocol. A C-arm image intensifier was used during the operation [13]. All cervical surgical instruments and the endoscopic system used were provided by Richard Wolf GmbH (Knittlingen, Germany) (Figure 2).

All APECD and PPECD procedures were performed by the same neurosurgeon with consistent patient positioning and endoscope techniques. For both approaches, patients were advised to wear a neck collar for 1 month after the operation to prevent recurrent disc herniation.

### 2.5. APECD

**Figure 3:**
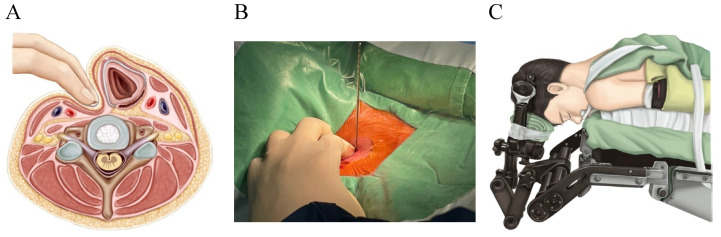
(**A**,**B**) The two-finger technique is shown above, with (**A**) a drawing of the technique on the left and (**B**) a photo of the technique being performed on the right. (**C**) Prone position with the neck slightly flexed, and the head fixed in the Mayfield head clamp.

The patient was positioned in a supine position under general anesthesia, with the neck kept in slight extension. The entire procedure was monitored using C-arm fluoroscopy. A two-finger technique was used to create a small, safe window between the lateral carotid artery and medial trachea-esophagus. The space allowed for the insertion of a thin dilator through the cervical fascia, anterior longitudinal ligament, and anterior annulus fibrosus between the bilateral longus colli muscles (Figure 3A,B). Depth was confirmed with the thin dilator, followed by insertion of a combined dilator-sleeve system. The thin dilator was removed and the working sleeve was positioned diagonally towards the intervertebral space.

During the operation, the operative sight was continuously irrigated with a saline solution using an irrigation system. Contralateral dissection of the uncinate process and posterior edge of the vertebral body was performed to aid in topographical orientation. Resection of bone using various instruments, such as a high-speed drill and manual reamer, were used to access the epidural space. The annulus fibrosus and posterior longitudinal ligament were opened to expose and excise the extruded disc material using a micro-punch, bone punch, and disc forceps. Coagulation and tissue ablation were performed using a high radiofrequency, low temperature bipolar probe (Elliquence, LLC, Baldwin, NY, USA) after confirming complete decompression [19].

### 2.6. PPECD

After induction of general anesthesia, the patient was positioned in a prone position with the neck slightly flexed, and the head fixed in a Mayfield head clamp (Figure 3C). The incision was determined based on anteroposterior (AP) view and lateral view C-arm fluoroscopy. A 7 mm transverse incision was made above the facet joint on the affected side, and a dilator was inserted until contact was made with bone on the zygapophyseal joint under lateral C-arm fluoroscopy. The working sleeve, featuring an oblique opening, was pushed through the dilator in a medial direction, and then the dilator was removed.

Under endoscopic visualization, the two adjacent vertebral laminae and the ipsilateral facet joint between them, forming a V-shape, were identified. An oval and diamond burr, or a bone punch were used to open the interlaminar window above the herniation, starting from the medial margin and extending towards the lateral margin, based on the location of the pathology.

The dural sac was visualized by partially removing the ligamentum flavum. To ensure a clear visual field and accurate identification of the nerves, the epidural venous plexus was coagulated using a high radiofrequency, low temperature bipolar probe, if needed. The nerves were carefully retracted, and a manual reamer was used to incise and remove the calcified disc and osteophytes. Then, the herniated disc was extracted with disc forceps. Once hemostasis was achieved, all instruments were removed [19,20].

### 2.7. Follow-Up

Postoperatively, a visual analogue scale (VAS) was used to gauge the levels of neck and arm pain. The neck disability index (NDI) and the modified MacNab criteria were also used as a measure of postoperative outcomes. The MacNab score was modified to incorporate the VAS score (post-PECD 1-year VAS versus pre-PECD VAS). The modified MacNab score for pain was defined as: excellent (no pain, VAS = 0 and 100% pain relief), good (50–99% pain relief), fair (1–49% pain relief), and poor (0% pain relief and no overall improvement) [21]. Plain lateral radiographs of the cervical spine in the neutral, flexion, and extension positions were obtained preoperative, and at 3 months and one year after the operation, and cervical MRI was performed one year after the operation.

### 2.8. Statistical Analysis

Continuous variables were presented as the median, 25th percentile, and 75th percentile, and categorical variables as count and percentage. The Mann–Whitney U test, Friedman test, and Wilcoxon signed ranks test were used for comparisons of continuous variables. Statistical analysis was conducted using the Statistical Package for the Social Sciences, version 22.0 (IBM Corp., Armonk, NY, USA). A value of *p* < 0.05 was considered to indicate statistical significance. Post-hoc analysis was conducted after the study has been completed and data have been collected.

## 3. Results

### 3.1. Surgical Findings and Postoperative Course

The surgical findings, operative time, and postoperative course of the patients are shown in Table 1. In the PPECD group, four patients had calcified discs, and another three were associated with ruptured discs. In the APECD group, all five patients had ruptured discs, and none were calcified. The median operative time of the APECD group was 88.0 min, and that of the PPECD group was 78.5 min (*p* = 0.088). The mean hospital stay was 2 days for both groups (*p* > 0.05). Both procedures were associated with minimal blood loss.

The postoperative complications are summarized in Table 2. There were no severe complications, such as tracheoesophageal injury, vascular injury, nerve or spinal cord injuries, prevertebral hematoma, swallowing dysfunction, or wound infection. Of the 43 patients, one patient had a durotomy without any long-term complications. Three patients reported experiencing upper limb dysesthesia for more than 3 months following the surgery. In these patients, only mild digital numbness remained as a symptom after one year. Two patients had temporary muscle weakness, which resolved within 6 months and one year, respectively. No complications associated with cerebrospinal fluid leakage, wound infection, poor healing, or permanent neurological deficits were observed. One patient in the PPECD group experienced neck and left arm pain, and a cervical MRI was performed 2 weeks after surgery. A comparison with the preoperative MRI performed at another hospital showed that the bulging disc and neuroforaminal narrowing were moderately improved, and no recurrent disc or postoperative hematoma was detected. The symptoms gradually resolved after 6 months of conservative treatment.

### 3.2. Recurrence or Revision Surgery

At 1 year follow-up, all patients remained free of symptoms and had not experienced any recurrences. None of the patients required revision surgery. However, after the one-year follow-up period, a patient who had previously undergone PPECD at the C5–C6 level in October 2019 had a recurrence of CDH involving the C5–C6 and C6–C7 levels in August 2023. This recurrence occurred after the patient remained asymptomatic until July 2023 when she received two treatments of a celiac plexus block for constipation while in a prolonged prone position. The potential link between this procedure and the symptom recurrence, 3 years after the initial PPECD surgery, required a revision PPECD in August 2023.

### 3.3. Clinical Outcomes

**Figure 4:**
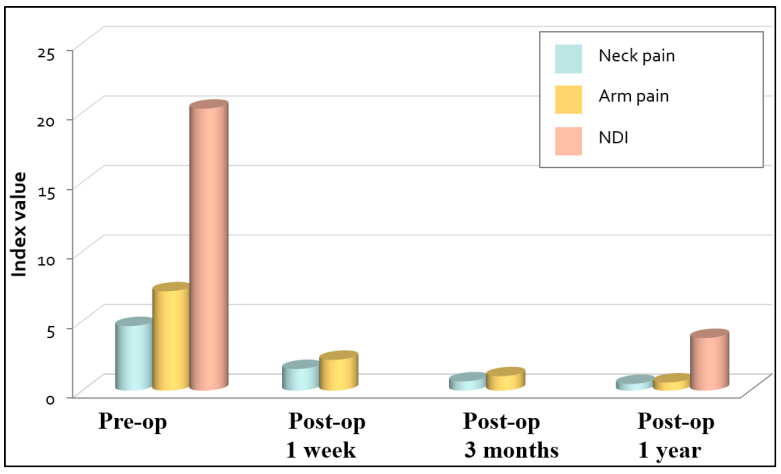
Mean visual analogue scale (VAS) scores for neck pain and arm pain, and Neck Disability Index. NDI = Neck Disability Index, pre-op = preoperative, post-op = postoperative. One of the 43 patients was lost to follow-up after 3 months postoperative.

**Figure 5:**
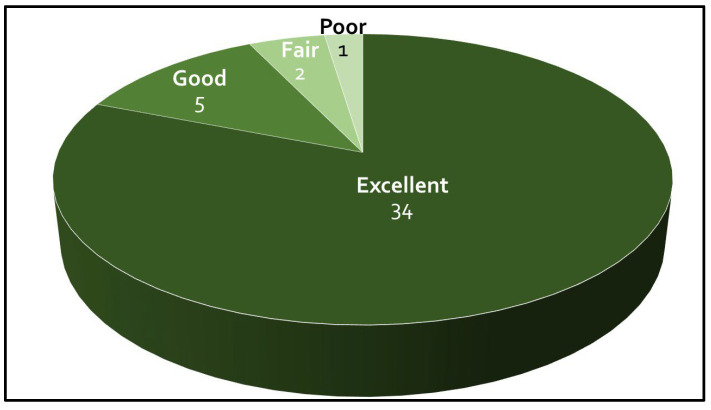
Modified MacNab score. One of the 43 patients was lost to follow-up after 3 months postoperative.

One of the 43 patients was lost to follow-up after 3 months postoperative. This patient demonstrated a moderate improvement of the VAS pain score at 3 months after surgery. The preoperative and postoperative VAS scores, NDI and modified MacNab scores of the 42 patients are presented in Figure 4 and Figure 5. There were no significant differences between the mean VAS pain score and NDI between the two groups (*p* = 0.561 and *p* = 0.906, respectively). Of the patients, 80.9% had excellent outcomes and 11.9% good outcomes. A fair outcome for relief of neck and arm pain was reported by 4.8% of the patients, and 2.4% reported a poor outcome.

### 3.4. Radiographic Outcomes

**Figure 6:**
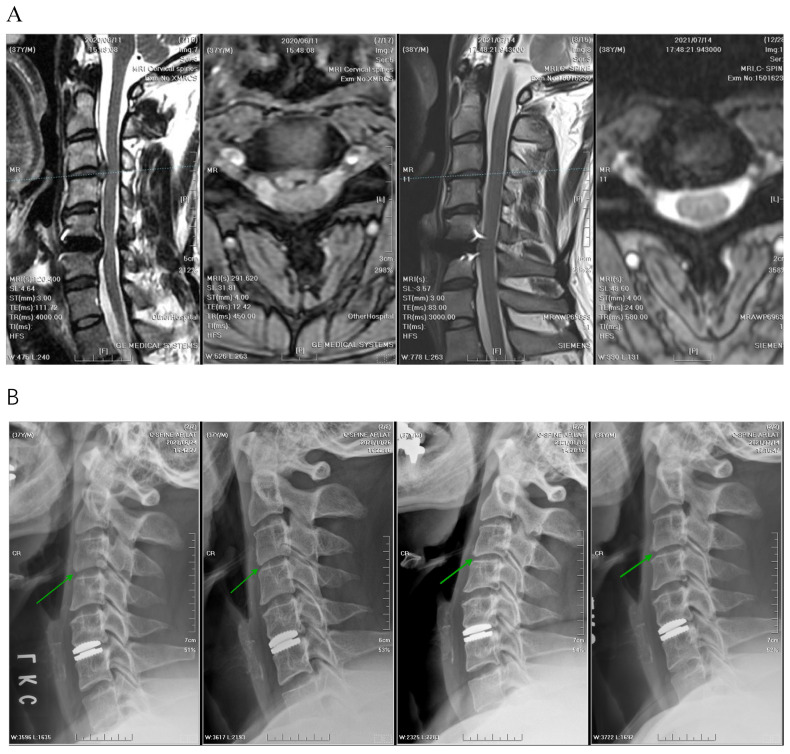
(**A**) The intervertebral disc height of C3–C4 decreased significantly from 5.0 mm to 3.2 mm before and after APECD (MRI before APECD on the left and one year after the surgery on the right). (**B**) The images from left to right are radiographs before APECD and 3 months, 6 months, and 1 year after the surgery. Green arrows point to the C3–C4 disc space. The reduction of disc height of C3–C4 was sufficient to be observed by the naked eye.

**Figure 7:**
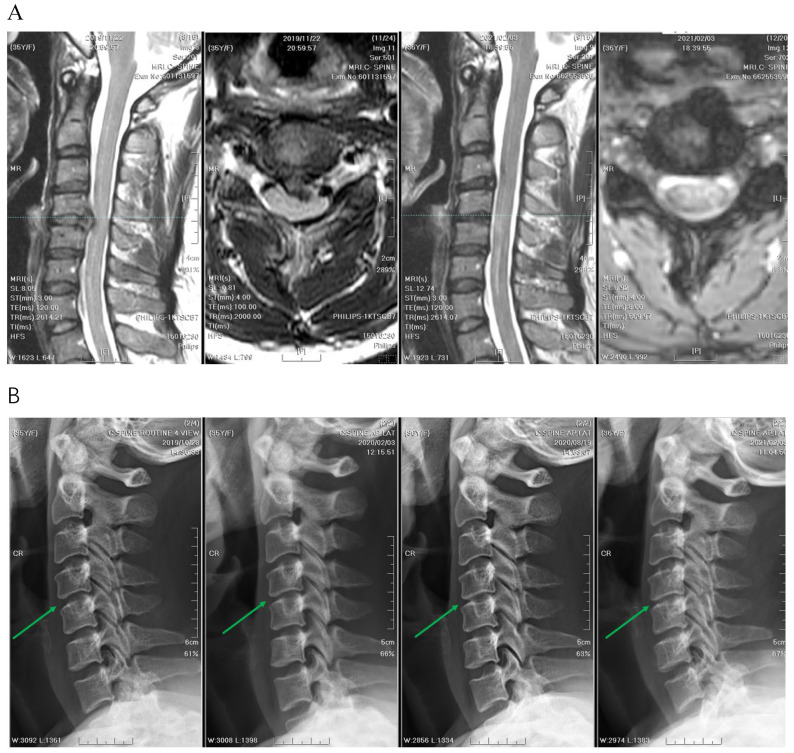
(**A**) The intervertebral disc height of C4–C5 decreased significantly from 4.4 mm to 3.8 mm, before and after APECD (MRI before APECD on the left and 1 year after the surgery on the right). (**B**) The images from left to right are radiographs before APECD, and 3 months, 6 months, and 1 year after the surgery. Green arrows point to the C4–C5 disc space. The reduction of disc height of C4–C5 was sufficient to be observed by the naked eye.

**Figure 8:**
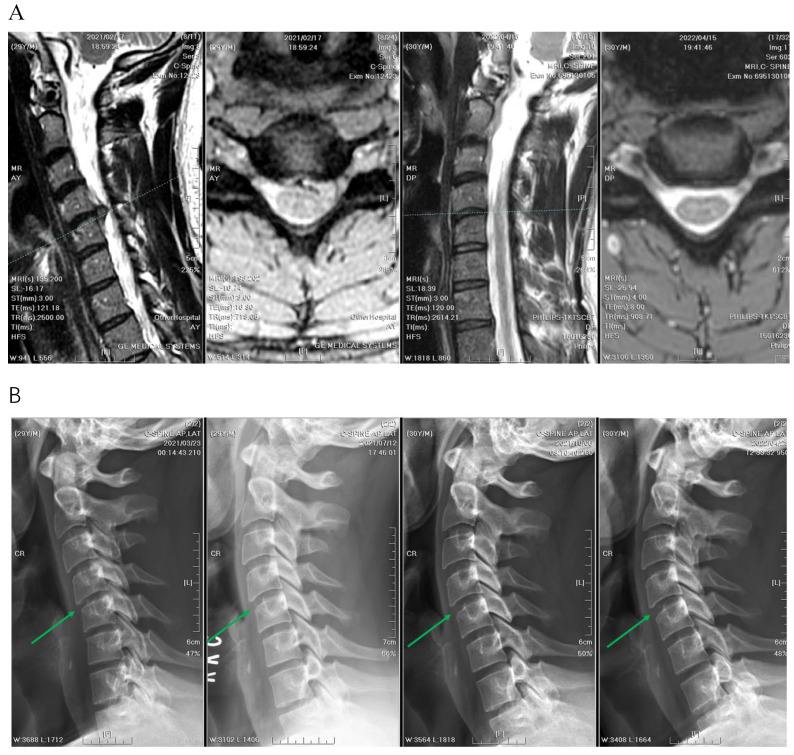
(**A**) The intervertebral disc height of C4–C5 decreased slightly from 4.4 mm to 4.1 mm, before and after PPECD (MRI before PPECD on the left and 1 year after the surgery on the right). (**B**) The images from left to right are radiographs before PPECD and 3 months, 6 months, and 1 year after the surgery. Green arrows point to the C4–C5 disc space.

A significant difference was observed in the median decrease in vertical disc height between the two groups (Figure 6, Figure 7 and Figure 8). The reduction in disc height measured using MRI was 1.3 mm in the APECD group, resulting in a decrease in median height from 5.03 mm to 3.73 mm. The reduction in disc height in the PPECD group was 0.3 mm (4.13 mm preoperative to 3.83 mm postoperative, *p* < 0.001) (Table 3). The slippage distance in flexion-extension did not show a significant change from the preoperative period to one-year postoperative.

**Figure 9:**
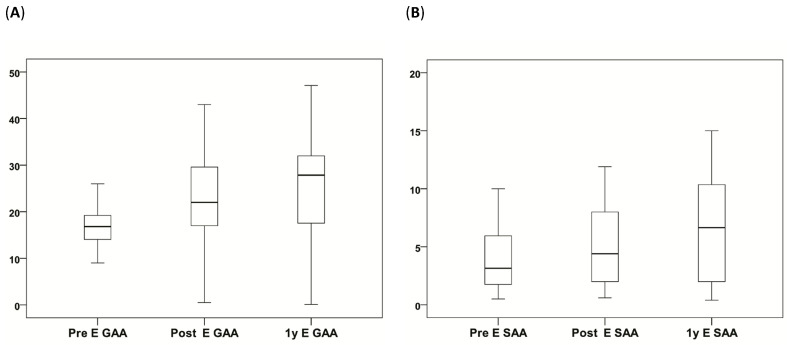
(**A**,**B**) The box plot displays the significant difference in E GAA and E SAA, as indicated in Table 4.

In the PPECD group, the LA was 5.55 preoperation, 3.10 three months postoperation, and 4.35 one-year postoperation (*p* = 0.068). The GAA in flexion of the PPECD group was 17.65 preoperation, 17.70 three months postoperation, and 18.80 one-year postoperation (*p* = 0.230). The SAA in flexion of the PPECD was 7.00 preoperation, 3.00 three months postoperation, and 5.60 one-year postoperation (*p* = 0.054). The GAA of the PPECD group in extension was 16.80 preoperation, 22.00 three months postoperation, and 27.85 one-year postoperation (*p* = 0.001). The SAA in extension of the PPECD group was 3.15 preoperation, 4.40 three months postoperation, and 6.65 one-year postoperation (*p* = 0.033). The GAA in extension was significantly different at 1 year after surgery compared to the preoperative value (*p* = 0.003). The SAA in extension was significantly different at 1 year after surgery compared to the preoperative value (*p* = 0.031). The SD of the PPECD group was 0.20 preoperation, 0.20 three months postoperation, and 0.20 one-year postoperation (*p* = 0.351) (Table 4 and Figure 9).

The LA of the APECD group was 2.20 preoperation, 2.50 three months postoperation, and 3.20 one-year postoperation (*p* = 0.549). The GAA in flexion of the APECD group was 16.90 preoperation, 17.40 three months postoperation, and 17.10 one-year postoperation (*p* = 0.819). The SAA in flexion of the APECD group was 3.50 preoperation, 4.80 three months postoperation, and 3.10 one-year postoperation (*p* = 0.247). The GAA in extension of the APECD group was 21.30 preoperation, 23.50 three months postoperation, and 21.20 one-year postoperation (*p* = 0.819). The SAA in extension of the APECD was 6.90 preoperation, 5.50 three months postoperation, and 4.20 one-year postoperation (*p* = 0.196). The SD of the APECD group was 0.20 preoperation, 0.30 three months postoperation, and 0.30 one-year postoperation (*p* = 0.247).

## 4. Discussion

In an era of minimally invasive surgery, PECD offers the advantages of targeted decompression with continuous visualization, thereby reducing operation-related trauma and optimizing function preservation. PECD serves as a bridge between conservative therapy and traditional surgery.

Our inclusion criteria are closely aligned with those used in other studies [7,22]. However, the number of patients who underwent APECD was significantly lower than that of patients who underwent PPECD due to the higher risk of intraoperative iatrogenic injury to tracheoesophageal and vascular structures. Because of the loose tissue distribution, access-related bleeding in APECD was relatively more difficult to control than in PPECD. There are significant differences observed between the two groups (5 vs. 38) in terms of median preoperative VAS score (arm pain) and ruptured disc. This discrepancy may be attributed to the necessity of considering the specific location of the pathology when selecting the surgical approach. An anterior cervical approach is generally favored for the removal of central disc herniations, which are often associated with ruptured discs. Consequently, the observed trend in patient distribution between the groups is likely an inherent outcome of these clinical considerations. Moreover, the observed differences between the patient groups may be attributed to the presence of ruptured discs and central disc herniations. Consequently, patients who underwent APECD are more likely to exhibit a more significant postoperative reduction in disc height compared to those who underwent PPECD.

In Taiwan, the average hospitalization period for ACDF is 4 or more days for over 90% of patients, as reported in Taiwan’s open claim database, the National Health Insurance Research Database (NHIRD) [23]. However, APECD and PPECD are associated with an average hospital stay of 2 days. It should be noted that the index date for PECD surgery is considered the first day, and the patient is discharged on the next day.

Our results showed that both approaches were associated with a significant decrease in the mean VAS pain scores. However, there was no notable difference in the mean VAS pain scores between the two procedures. (*p* = 0.561 for neck pain and *p* = 1.000 for arm pain). In terms of the NDI, both groups had a high proportion of patients with an excellent or good outcome; however, the difference between the groups was not statistically significant (*p* = 0.132). According to the modified MacNab scores [21], approximately 93% of patients reported an excellent or good outcomes for relief of neck and arm pain during the follow-up period.

Three patients had upper limb dysesthesia for more than 3 months following the surgery, and two patients had temporary muscle weakness. These symptoms were not found to be related to calcified or ruptured discs, and resolved gradually with conservative treatment.

In the prospective study conducted by Ruetten et al. [11], the 2-year postoperative recurrence rate after APECD was 3.7%. Similarly, in another study by Ruetten et al. [7] which involved a 2-year postoperative follow-up of 87 patients who underwent PPECD, the recurrence rate was low (3.4%), and the success rate was deemed satisfactory (96%). In line with previous studies, our recurrence rate was 0% one year after surgery. This may be related to the minimally invasive opening of the annulus fibrosus and the requirement to wear a neck collar for 1 month after surgery. Notably, there was one recurrence 3 years after the procedure.

Our results showed there was no significant change in the SD in flexion–extension from preoperative to 1 year after surgery. The disc height reduction was greater in the APECD group, which is likely due to the removal of more disc tissue during the procedure, and this result is consistent with that of previous studies [3,7,11,23,24]. Computed tomography (CT) is indeed a suitable method for measuring disc height. However, we did not utilize CT in our study primarily due to incomplete CT records. The incompleteness is due to our primary focus during annual follow-up visits being on assessing whether there is any recurrent neural compression. Additionally, cost considerations and the policies of the National Health Insurance system in Taiwan also influenced our decision not to use CT for measuring disc height. Despite the larger reduction in disc height, both groups showed significant clinical improvement. This lack of significant change in the SD in flexion-extension may be attributed to the effective decompression of the nerve root and/or spinal cord, achieved with minimal damage to ligamentous and bony structures during the surgical procedure.

Notably, in the PPECD group one patient experienced a significant narrowing of the anterior disc space after surgery. A possible reason for this may be the patient’s high body weight, which exceeded 100 kg, along with her height of 176.5 cm. The patient’s body mass index (BMI) was 33.5 kg/m^2^, which is relatively high and uncommon among Taiwanese individuals.

There was a notable disparity observed in the GAA and SAA in extension between preoperation and one-year postoperation in the PPECD group (*p* = 0.003 and 0.031, respectively) (Table 4). In the APECD group there were no significant differences in GAA and SAA during extension. This outcome may be linked to preoperative painful disability and a propensity for flexion, both of which improved after the operation. The absence of this finding in the APECD group may be attributed to the small number of patients.

Ren et al. [25] reported that the postoperative stress distribution after PPECD is uniform. Three-dimensional finite element analysis also demonstrated that the postoperative stability of PPECD is superior to that of ACDF, particularly when performing rear protraction and lateral flexion. This conclusion is consistent with the findings of the current study, where the difference in SD between one year after the operation and preoperation was not statistically significant, indicating that the stability was not affected during the one-year period, despite the observed increase in angulation in extension.

### Limitations

The measurements in our study were from lateral radiographs, and may have been affected by variables such as radiographic technique, patient positioning, and the anatomical region captured, such as in three patients with the index level at C7–T1, which were not clearly depicted by plain radiography. We acknowledge the limitations of our study, including the decision not to use CT for measuring disc height. This choice was made due to the constraints of the National Health Insurance system in Taiwan, which does not allow for the simultaneous use of MRI and CT in follow-up assessments. To prioritize patient benefit, we opted to use MRI as the primary imaging modality for follow-up. The potential sources of bias in our study include the small sample size of APECD cases, which limited our ability to use mean values and instead necessitated the use of median values. The single-center nature of our study and the relatively small sample size, along with the resultant demographic imbalances, are significant limitations.

## 5. Conclusions

While ACDF is considered the gold standard for the treatment of cervical spondylotic radiculopathy, it is noteworthy that the clinical outcomes after PECD were not compromised by loss of disc height.

This study provides evidence that PECD is an effective approach for managing CDH with or without bony involvement. The careful selection of patients and meticulous surgical technique are crucial factors in mitigating the incidence of complications.

Treating CDH with APECD or PPECD is feasible with appropriate patient selection, and the procedures effectively relieve pain, numbness, and reduce disability. Stability was not affected during the first year after surgery, despite the observed increase in angulation in extension. However, there was a statistically significant decrease in disc height in patients who underwent APECD compared to those who underwent PPECD. Although the disc space was significantly reduced after APECD, the symptoms were greatly improved and no new symptoms occurred.

## Figures and Tables

**Table 1:** Patient baseline characteristics, operative time, and postoperative course.

Characteristics	APECD	PPECD	*p* Value
OP age (*n* = 43)	48 (36.0–59.5)	48 (40.3–53.0)	0.936
Sex (*n* = 43)			0.362
Female	3 (60.0%)	14 (36.8%)	
Male	2 (40.0%)	24 (63.2%)	
Median preoperative VAS score (neck pain)	4.0 (4.0–5.0)	5.0 (4.0–5.0)	0.510
Median preoperative VAS score (arm pain)	6.0 (6.0–6.5)	7.0 (7.0–8.0)	0.013 *
Median operative time	88.0 (84.0–130.0)	78.5 (68.8–95.0)	0.088
Median hospital stay	2.0 (2.0–2.0)	2.0 (2.0–2.0)	0.604
Blood loss	minimal	minimal	--
Ruptured disc	5 (100.0%)	3 (7.9%)	<0.001 **
Calcified disc	0 (0.0%)	4 (10.5%)	1.000
Shoulder approach	--	14 (36.8%)	--
Axillary approach	--	24 (63.2%)	--

Fisher’s exact test or Mann–Whitney U test, Median (IQR). * *p* < 0.05, ** *p* < 0.01.

**Table 2:** Complications of patients undergoing APECD and PPECD.

Total Complications	APECD	PPECD
Tracheoesophageal injury	0	0
Vascular injury	0	0
Nerve or spinal cord injury	0	0
Temporary headache	0	1
Temporary muscle weakness	0	2
Upper limb dysesthesia > 3 months	0	3
Dura tear	0	1
Wound infection	0	0
Repeat surgery	0	0

**Table 3:** Preoperative and postoperative disc height of the APECD and PPECD groups.

	APECD (*n* = 5)	PPECD (*n* = 37)	*p* Value
Pre-op average DH of index segment	50.3 (45.8, 57.7)	41.3 (37.0, 44.9)	0.003 **
Post-op average DH of index segment	37.3 (33.8, 44.7)	38.3 (34.4, 41.6)	0.991
Post-op average DH of index segment − Pre-op average DH of index segment	−13.00 (−17.17, −7.83)	−3.00 (−3.67, −1.08)	<0.001 **

Pre-op = preoperative, post-op = postoperative, DH = disc height. Mann–Whitney U test, median (IQR). ** *p* < 0.01.

**Table 4:** Differences in angles between time points for APECD and PPECD.

	*n*	Preoperative	3 Months Postoperative	1 Year Postoperative	*p* Value
APECD					
LA	5	2.20 (1.20, 3.55)	2.50 (1.35, 5.50)	3.20 (1.80, 4.10)	0.549
F GAA	5	16.90 (15.15, 19.00)	17.40 (15.75, 19.20)	17.10 (16.15, 19.20)	0.819
F SAA	5	3.50 (1.90, 5.05)	4.80 (1.85, 5.35)	3.10 (1.35, 4.30)	0.247
E GAA	5	21.30 (18.05, 27.90)	23.50 (20.70, 28.25)	21.20 (15.80, 28.50)	0.819
E SAA	5	6.90 (4.55, 7.30)	5.50 (3.05, 6.70)	4.20 (1.90, 5.35)	0.196
SD	5	0.20 (0.19, 0.32)	0.30 (0.19, 0.40)	0.30 (0.17, 0.46)	0.247
PPECD					
LA	34#	5.55 (2.68, 8.00)	3.10 (2.00, 5.00)	4.35 (2.03, 9.13)	0.068
F GAA	34#	17.65 (15.48, 19.63)	17.70 (15.85, 20.93)	18.80 (16.35, 20.68)	0.230
F SAA	34#	7.00 (4.70, 10.00)	3.00 (1.80, 7.00)	5.60 (4.20, 8.00)	0.054
E GAA	34#	16.80 (13.58, 19.55)	22.00 (17.00, 29.80)	27.85 (17.33, 32.00)	0.001 **^†^
E SAA	34#	3.15 (1.63, 5.98)	4.40 (2.00, 8.00)	6.65 (2.00, 10.53)	0.033 *^†^
SD	34#	0.20 (0.10, 0.34)	0.20 (0.11, 0.30)	0.20 (0.13, 0.35)	0.351

*n* = patient number, LA = lordotic angle, F = flexion, E = extension, GAA = global alignment angle, SAA = segmental alignment angle, SD = slippage distance. ^†^ Post-hoc analysis (Bonferroni), E GAA pre-op vs. post-op 3 mon, *p* = 0.003; E GAA pre-op vs. post-op 1 year, *p* = 0.003; E SAA pre-op vs. post-op 1 year, *p* = 0.031. # Three patients with the index level at C7–T1, which were not clearly depicted on plain radiographs, were not included. Friedman test. * *p* < 0.05, ** *p* < 0.01.

## Data Availability

All data generated or analyzed in this study have been included in this article. For further inquiries, please contact the corresponding authors.

## Abbreviations

ACDF	anterior cervical discectomy and fusion
APECD	anterior approach of PECD
CDH	cervical disc herniation
CT	computed tomography
GAA	global alignment angle
LA	lordotic angle
MRI	magnetic resonance imaging
NDI	neck disability index
PECD	percutaneous endoscopic cervical discectomy
PPECD	posterior approach of PECD
ROM	range of motion
SAA	segmental alignment angle
SD	slippage distance
VAS	visual analogue scale

## References

[1] Heary R.F., Ryken T.C., Matz P.G., Anderson P.A., Groff M.W., Holly L.T., Kaiser M.G., Mummaneni P.V., Choudhri T.F., Vresilovic E.J. (2009). Cervical laminoforaminotomy for the treatment of cervical degenerative radiculopathy. J. Neurosurg. Spine.

[2] Chiu J.C., Clifford T.J., Greenspan M., Richley R.C., Lohman G., Sison R.B. (2000). Percutaneous microdecompressive endoscopic cervical discectomy with laser thermodiskoplasty. Mt. Sinai J. Med..

[3] Ahn Y., Lee S.H., Shin S.W. (2005). Percutaneous endoscopic cervical discectomy: Clinical outcome and radiographic changes. Photomed. Laser Surg..

[4] Nakamura S., Taguchi M. (2018). Percutaneous Endoscopic Cervical Discectomy: Surgical Approaches and Postoperative Imaging Changes. Asian Spine J..

[5] Wan Q., Zhang D., Li S., Liu W., Wu X., Ji Z., Ru B., Cai W. (2018). Posterior percutaneous full-endoscopic cervical discectomy under local anesthesia for cervical radiculopathy due to soft-disc herniation: A preliminary clinical study. J. Neurosurg. Spine.

[6] Zhang J., Zhou Q., Yan Y., Ren J., Wei S., Zhu H., Song Z. (2022). Efficacy and safety of percutaneous endoscopic cervical discectomy for cervical disc herniation: A systematic review and meta-analysis. J. Orthop. Surg. Res..

[7] Ruetten S., Komp M., Merk H., Godolias G. (2007). A new full-endoscopic technique for cervical posterior foraminotomy in the treatment of lateral disc herniations using 6.9-mm endoscopes: Prospective 2-year results of 87 patients. Minim. Invasive Neurosurg..

[8] Ye Z.Y., Kong W.J., Xin Z.J., Fu Q., Ao J., Cao G.R., Cai Y.Q., Liao W.B. (2017). Clinical Observation of Posterior Percutaneous Full-Endoscopic Cervical Foraminotomy as a Treatment for Osseous Foraminal Stenosis. World Neurosurg..

[9] Burkhardt B.W., Müller S., Oertel J.M. (2016). Influence of Prior Cervical Surgery on Surgical Outcome of Endoscopic Posterior Cervical Foraminotomy for Osseous Foraminal Stenosis. World Neurosurg..

[10] Oertel J.M., Philipps M., Burkhardt B.W. (2016). Endoscopic Posterior Cervical Foraminotomy as a Treatment for Osseous Foraminal Stenosis. World Neurosurg..

[11] Ruetten S., Komp M., Merk H., Godolias G. (2008). Full-endoscopic cervical posterior foraminotomy for the operation of lateral disc herniations using 5.9-mm endoscopes: A prospective, randomized, controlled study. Spine.

[12] Tzaan W.C. (2011). Anterior percutaneous endoscopic cervical discectomy for cervical intervertebral disc herniation: Outcome, complications, and technique. J. Spinal Disord. Tech..

[13] Komp M., Oezdemir S., Hahn P., Ruetten S. (2018). Full-endoscopic posterior foraminotomy surgery for cervical disc herniations. Oper. Orthop. Traumatol..

[14] Yang J.S., Chu L., Chen L., Chen F., Ke Z.Y., Deng Z.L. (2014). Anterior or posterior approach of full-endoscopic cervical discectomy for cervical intervertebral disc herniation? A comparative cohort study. Spine.

[15] Chen T.Y., Chen W.H., Tzeng C.Y., Huang C.W., Yang C.C., Chen H.T., Chang C.C., Lee C.Y., Tsou H.K. (2020). Anterior bone loss after cervical Bryan disc arthroplasty: Insight into the biomechanics following total disc replacement. Spine J..

[16] Harrison D.E., Harrison D.D., Cailliet R., Troyanovich S.J., Janik T.J., Holland B. (2000). Cobb method or Harrison posterior tangent method: Which to choose for lateral cervical radiographic analysis. Spine.

[17] Yang C.-C., Chen T.-Y., Chen W.-H., Tzeng C.-Y., Huang C.-W., Lin R.-H., Kao T.-H., Chen H.-T., Chang C.-C., Tsou H.-K. (2023). Anterior Bone Loss after Cervical Baguera C Disc versus Bryan Disc Arthroplasty. BioMed Res. Int..

[18] Hou G.L., Chen C.M., Chen K.T., Xu S.E., Tao L., Kong L.T., Lai G.Z., Shi L., Chu L., Chen Y.D. (2022). Circumferential Decompression Technique of Posterior Endoscopic Cervical Foraminotomy. Biomed. Res. Int..

[19] VERTEBRIS Cervical Brochure—Richard Wolf—Catalogs. https://pdf.medicalexpo.com/pdf/richard-wolf/vertebris-cervical-brochure/78958-180036.html#open.

[20] Hsiao M.C., Chung K.C., Tsou H.K., Chang Y.H., Kao T.H., Chang C.P. (2024). Cervical Conjoined Nerve Root During Posterior Percutaneous Endoscopic Cervical Discectomy. World Neurosurg..

[21] Shen S.C., Chen H.C., Tsou H.K., Lin R.H., Shih Y.T., Huang C.W., Tang C.L., Chen H.T., Chang C.C., Tzeng C.Y. (2023). Percutaneous endoscopic lumbar discectomy for L5-S1 disc herniation based on image analysis and clinical findings: A retrospective review of 345 cases. Medicine.

[22] Ruetten S., Komp M., Merk H., Godolias G. (2009). Full-endoscopic anterior decompression versus conventional anterior decompression and fusion in cervical disc herniations. Int. Orthop..

[23] Chen Y.C., Wu J.C., Chang H.K., Huang W.C. (2019). Early Discharge for Anterior Cervical Fusion Surgery: Prediction of Readmission and Special Considerations for Older Adults. Int. J. Environ. Res. Public Health.

[24] Kim C.H., Chung C.K., Kim H.J., Jahng T.A., Kim D.G. (2009). Early outcome of posterior cervical endoscopic discectomy: An alternative treatment choice for physically/socially active patients. J. Korean Med. Sci..

[25] Ren J., Li R., Zhu K., Han X., Liu X., He Y., Sun Z. (2019). Biomechanical comparison of percutaneous posterior endoscopic cervical discectomy and anterior cervical decompression and fusion on the treatment of cervical spondylotic radiculopathy. J. Orthop. Surg. Res..

